# Friction Matches

**Published:** 1873-02

**Authors:** 


### FRICTION MATCHES

Often throw out a disagreeable, unhealthy odor. Families can make a safe and almost odorless match for themselves, at a very trifling cost, thus: mix together thirty-four parts of phosphorus; fifty parts of nitrate of potash; twenty-six parts of chlorate of potash; forty-eight parts of red lead, and forty-two parts of best glue.

